# Significance of the *BRAF* mRNA Expression Level in Papillary Thyroid Carcinoma: An Analysis of The Cancer Genome Atlas Data

**DOI:** 10.1371/journal.pone.0159235

**Published:** 2016-07-13

**Authors:** Young Jun Chai, Jin Wook Yi, Hyeon-Gun Jee, Young A Kim, Ju Han Kim, Mingzhao Xing, Kyu Eun Lee

**Affiliations:** 1 Department of Surgery, Seoul National University Boramae Medical Center, 20 Boramae-ro 5-gil, Dongjak-gu, Seoul, 156–70, Korea; 2 Cancer Research Institute, Seoul National University College of Medicine, 101 Daehak-ro, Jongno-gu, Seoul, 110–744, Korea; 3 Department of Surgery, Seoul National University Hospital and College of Medicine, 101 Daehak-ro, Jongno-gu, Seoul, 110–744, Korea; 4 Center for Chronic Disease, Research Institute, National Medical Center, Seoul, Korea; 5 Department of Pathology, Seoul National University Boramae Medical Center, 20 Boramae-ro 5-gil, Dongjak-gu, Seoul, 156–70, Korea; 6 Division of Biomedical Informatics, Systems Biomedical Informatics Research Center, Seoul National University College of Medicine, 101 Daehak-ro, Jongno-gu, Seoul, 110–744, Korea; 7 Division of Endocrinology and Metabolism, Johns Hopkins University School of Medicine, 1830E Monument St, Ste 333, Baltimore, Maryland, 21287, United States of America; IPATIMUP/Faculty of Medicine of the University of Porto, PORTUGAL

## Abstract

**Background:**

*BRAF*^V600E^ is the most common mutation in papillary thyroid carcinoma (PTC), and it is associated with high-risk prognostic factors. However, the significance of the *BRAF* mRNA level in PTC remains unknown. We evaluated the significance of *BRAF* mRNA expression level by analyzing PTC data from The Cancer Genome Atlas (TCGA) database.

**Methods:**

Data from 499 patients were downloaded from the TCGA database. After excluding other PTC variants, we selected 353 cases of classic PTC, including 193 cases with *BRAF*^V600E^ and 160 cases with the wild-type *BRAF*. mRNA abundances were measured using RNA-Seq with the Expectation Maximization algorithm.

**Results:**

The mean *BRAF* mRNA level was significantly higher in *BRAF*^V600E^ patients than in patients with wild-type *BRAF* (197.6 vs. 179.3, *p* = 0.031). In wild-type *BRAF* patients, the mean *BRAF* mRNA level was higher in cases with a tumor > 2 cm than those with a tumor ≤ 2.0 cm (189.4 vs. 163.8, *p =* 0.046), and was also higher in cases with lymph node metastasis than in those without lymph node metastasis (188.5 vs. 157.9, *p =* 0.040). Within *BRAF*^V600E^ patients, higher *BRAF* mRNA expression was associated with extrathyroidal extension (186.4 vs. 216.4, *p =* 0.001) and higher T stage (188.1 vs. 210.2, *p =* 0.016).

**Conclusions:**

A higher *BRAF* mRNA expression level was associated with tumor aggressiveness in classic PTC regardless of *BRAF* mutational status. Evaluation of *BRAF* mRNA level may be helpful in prognostic risk stratification of PTC.

## Introduction

Thyroid carcinoma is the most common endocrine malignancy, and its incidence has increased rapidly over the past few decades. In 2014, an estimated 62,980 new patients were expected in the United States [1]. Fortunately, the prognosis is generally excellent, and the thyroid cancer mortality is as low as 0.5 cases per 100,000 people [2]. However, a subset of thyroid carcinomas has a poor prognosis that is not adequately explained by traditional staging systems. Recent advances in cancer genetics provide new opportunities for improved assessment, and the molecular markers now available represent an effective strategy for the diagnosis and prognostication of thyroid carcinoma.

*BRAF* somatic mutations, the most extensively investigated molecular markers, are the most common genetic alterations in papillary thyroid carcinoma (PTC). One somatic mutation, *BRAF*^V600E^, results in the substitution of a valine by a glutamate at reside 600 and is associated with adverse prognostic factors such as extrathyroidal extension (ETE), lymph node metastasis, advanced American Joint Committee on Cancer (AJCC) stage, recurrence, distant metastasis, and poor survival [3–6]. This is explained by the fact that *BRAF*^V600E^ mutation, via activation of the MAP kinase pathway, causes loss of expression of thyroid genes and refractoriness to radioiodine, as well as up-regulation of angiogenic and tumor-promoting molecules [7, 8]. On the other hand, the incidence of the *BRAF*^V600E^ mutation in classic PTC varies widely between studies (38–83%) [9], and its positive predictive value for cancer recurrence is only 28% [10]. Furthermore, the results of several studies have challenged the notion that the *BRAF*^V600E^ mutation is a valuable prognostic marker [11, 12]. Consequently, the prognostic use of the *BRAF*^V600E^ mutation may not be generalizable to all clinical settings.

Several studies have suggested that the behavior of PTC is influenced not only by *BRAF* mutation status, but also by the expression level of the Braf mutant protein or the percentage of *BRAF* mutant alleles. One study reported that higher expression of Braf mutant protein predicts aggressive tumor behavior in PTC [13]. Other studies assessed the correlation between the percentage of *BRAF* mutant alleles and clinical outcomes in PTC using pyrosequencing [14, 15]. The results showed that higher percentages of *BRAF* mutant alleles were associated with poor prognostic factors such as older age, larger tumor size, ETE, and higher recurrence rate.

In this study, we analyzed the *BRAF* mRNA expression level in cases of PTC from The Cancer Genome Atlas (TCGA) database to investigate the significance of the level of *BRAF* mRNA expression in PTC and evaluate its prognostic value.

## Materials and Methods

### Data acquisition

As of March 2015, TCGA group made available multiple types of genomic data regarding thyroid carcinoma, including somatic mutation, exome sequencing, methylation array, mRNA expression count, microRNA expression count and clinical information. We downloaded the data on somatic mutation, mRNA expression count, and clinical information from the TCGA data portal (https://tcga-data.nci.nih.gov/tcga/tcgaDownload.jsp). All patient information was anonymized and de-identified in this database. According to TCGA publication guidelines (http://cancergenome.nih.gov/publications/publicationguidelines), there are no restrictions on the publication of these somatic mutation and mRNA sequencing data and no specific permission is required for investigators to publish papers containing or referring to these data. Somatic mutation data were provided as a mutation call file by the Broad Institute and the Baylor College of Medicine. The Illumina Genome Analyzer was used as the platform for DNA sequencing (Illumina Inc., San Diego, CA, USA). mRNA sequencing data, obtained by Illumina HiSeq 2000 RNA Sequencing Version 2 analysis, were provided by the University of North Carolina. mRNA expression counts were obtained via the TCGA portal and are expressed as RNA-Seq by Expectation Maximization (RSEM) values. RSEM is an accurate software tool for quantifying transcript abundances from RNA-Seq data [16].

After excluding the patients with missing information, we downloaded data from a total of 499 patients. Two authors (YJC, JWY) independently reviewed every scanned original pathologic report file and revised incorrect or missing clinical information. In cases of multifocal PTC, the largest tumor was analyzed.

### Patient selection

Of the 499 cases of PTC, 101 cases of follicular variant, 35 cases of tall cell variant, and eight cases of other variants were excluded, leaving a total of 355 classic PTCs. Subtype classification of the PTC patients was based on a previously published paper by Cancer Genome Atlas Research Network [17]. Follicular variant or tall cell variant PTC was diagnosed when more than 99% of the tumor exhibited a follicular pattern or more than 50% of tall cell features, respectively [17]. Two patients with *BRAF* mutations other than V600E (N581T, V459V) were also excluded. Ultimately, 193 classic PTCs with *BRAF*^V600E^ and 160 classic PTCs with wild-type *BRAF* were selected for the analysis.

### Statistics

Data were analyzed using the R software version 3.0.2 (R Foundation for Statistical Computing, Vienna, Austria). Chi-square tests and Fisher’s exact test were used to compare categorical variables. Unpaired two sample t-test and linear regression analysis were performed to compare mRNA expression counts. Recursive Partitioning and Regression Trees were used to calculate the cut-off value of *BRAF*^V600E^ mRNA expression above which the expression levels correlate with poor prognosis parameters. The Kaplan-Meier estimator was used for survival analysis. A *p*-value < 0.05 was considered significant.

## Results

**Fig 1:**
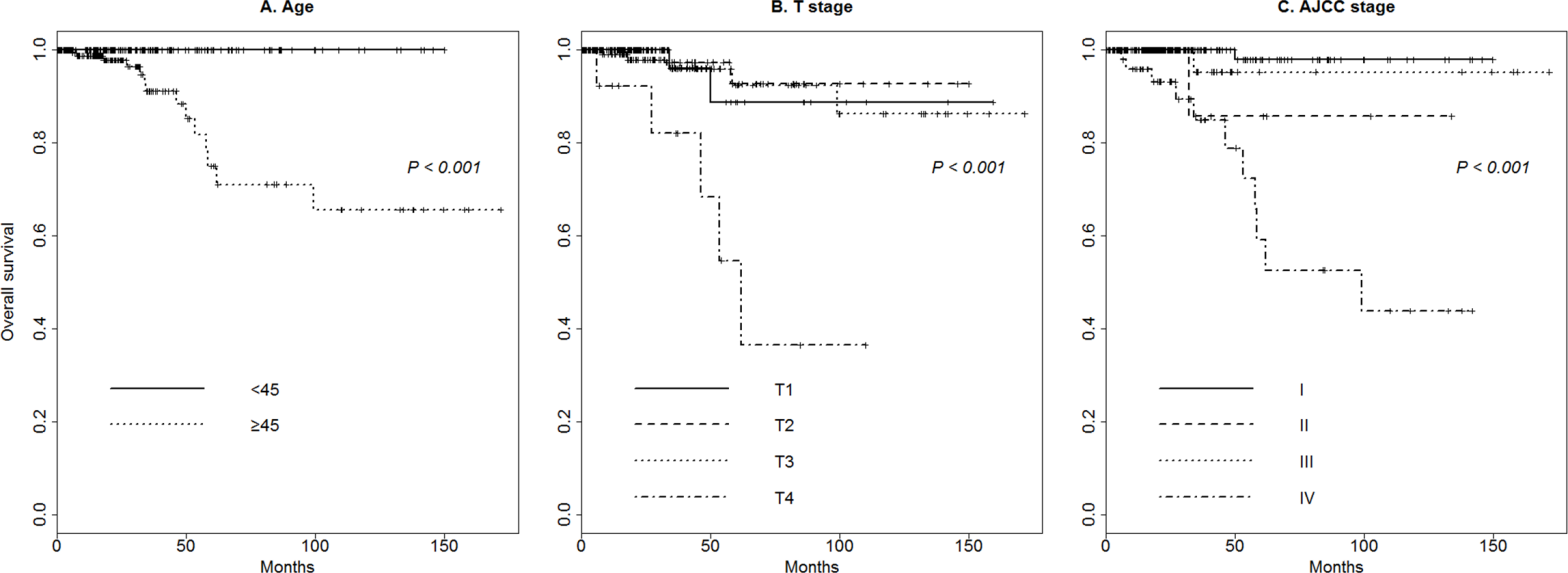
Overall survival of classic papillary thyroid carcinoma patients. (A) Age ≥ 45, (B) higher Tumor stage (T stage), and (C) higher American Joint Committee on Cancer (AJCC) stage were associated with poorer overall survival.

The median follow-up duration was 20.7 months (range, 0.03–171.70 months). Fig 1 shows Kaplan–Meier overall survival for the study population based on clinicopathological characteristics. Age ≥ 45 (*p* < 0.001), higher Tumor stage (T stage, *p* < 0.001), and higher AJCC stage (*p* < 0.001) were associated with poorer overall survival. Other clinicopathological characteristics such as gender (*p =* 0.293), size > 2.0 cm (*p =* 0.186), ETE (*p =* 0.14), presence of thyroiditis (*p =* 0.129), and lymph node metastasis (*p =* 0.225) were not significantly associated with overall survival.

### *BRAF* mutation status and clinicopathological characteristics

Clinicopathological characteristics of classic PTC patients classified according to *BRAF* mutation status are shown in Table 1. None of the factors, including age, gender, thyroiditis, ETE, tumor size, AJCC stage, recurrence, or vital status, differed significantly between the *BRAF*^V600E^ and wild-type *BRAF* patients.

**Table 1:** Clinicopathological characteristics of classic papillary thyroid carcinoma patients with *BRAF*^V600E^ or wild-type *BRAF*.

	Wild-type *BRAF* (n = 160)	*BRAF*^V600E^ (n = 193)	p—value
**Age**			
< 45 years	78 (48.8%)	92 (49.7%)	0.924
≥ 45 years	82 (51.2%)	101 (52.3%)	
**Gender**			
Male	41 (25.6%)	55 (28.5%)	0.629
Female	119 (74.4%)	138 (71.5%)	
**Thyroiditis**			
No	47 (29.4%)	65 (33.7%)	1.000
Yes	49 (30.6%)	67 (34.7%)	
n.a	64 (40.0%)	61 (31.6%)	
**Extrathyroidal extension**			
No	115 (71.9%)	121 (62.7%)	0.087
Yes	45 (28.1%)	72 (37.3%)	
**Tumor size**			
≤ 2.0 cm	63 (39.4%)	83 (43.0%)	0.561
> 2.0 cm	97 (60.6%)	110 (57.0%)	
**T stage**			
T1, T2	102 (63.8%)	110 (57.0%)	0.238
T3, T4	58 (36.2%)	83 (43.0%)	
**N stage**			
N0	44 (27.5%)	63 (32.6%)	0.572
N1	84 (52.5%)	93 (48.2%)	
Nx	32 (20.0%)	37 (19.2%)	
**AJCC stage**			
I, II	104 (65.0%)	129 (66.8%)	0.802
III, IV	56 (35.0%)	64 (33.2%)	
**Recurrence**			
No	149 (93.1%)	176 (91.2%)	0.637
Yes	11 (6.9%)	17 (8.8%)	
**Vital Status**			
Alive	155 (96.9%)	184 (95.3%)	0.643
Dead	5 (3.1%)	9 (4.7%)	

Abbreviations: AJCC = American Joint Committee on Cancer

### *BRAF* mRNA expression levels in *BRAF*^V600E^ and wild-type *BRAF* classic PTC

**Fig 2:**
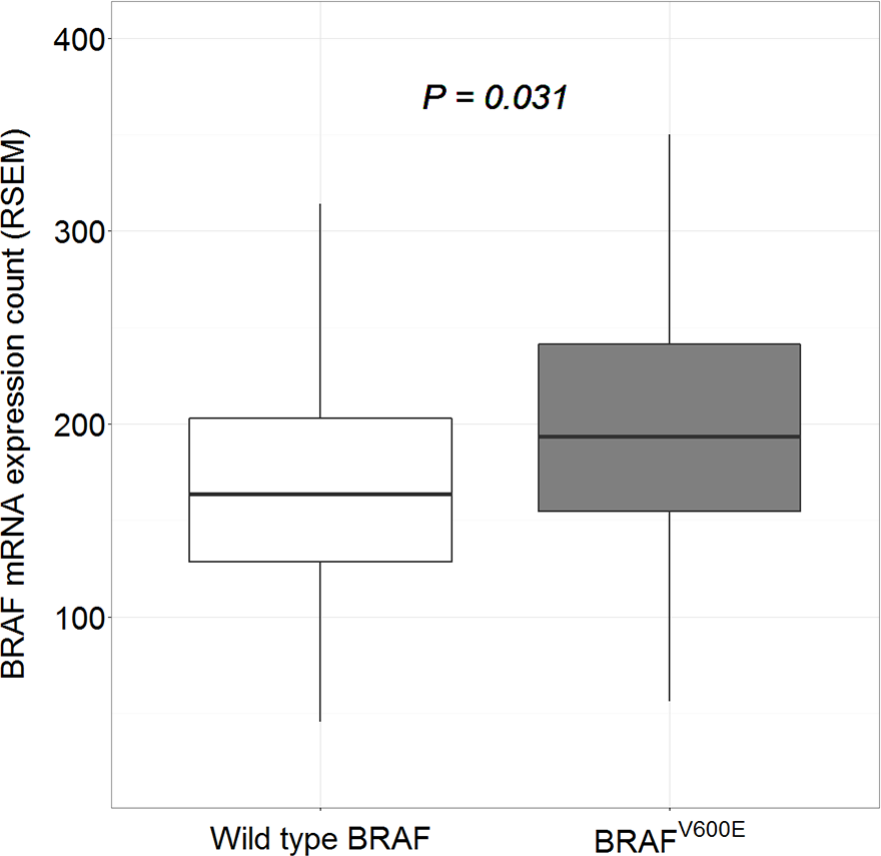
*BRAF* mRNA expression counts in classic papillary thyroid carcinoma patients. The mean *BRAF* mRNA expression count was significantly higher in *BRAF*^V600E^ patients than in patients with wild-type *BRAF*.

The *BRAF* mRNA expression counts for the study patients are shown in S1 Table. The count data for the wild-type *BRAF* and *BRAF*^V600E^ patients can be summarized as follows: for wild-type *BRAF* patients, minimum 45.3, lower quartile 128.7, median 166.2, mean 179.3 and upper quartile 204.2; for *BRAF*^V600E^ patients, minimum 56.1, lower quartile 154.7, median 194.1, mean 197.6, and upper quartile 243.5 (Fig 2). The mean *BRAF* mRNA expression count was significantly higher in *BRAF*^V600E^ patients than in patients with wild-type *BRAF* (197.6 vs. 179.3, *p* = 0.031).

### Significance of the *BRAF* mRNA expression level

**Fig 3:**
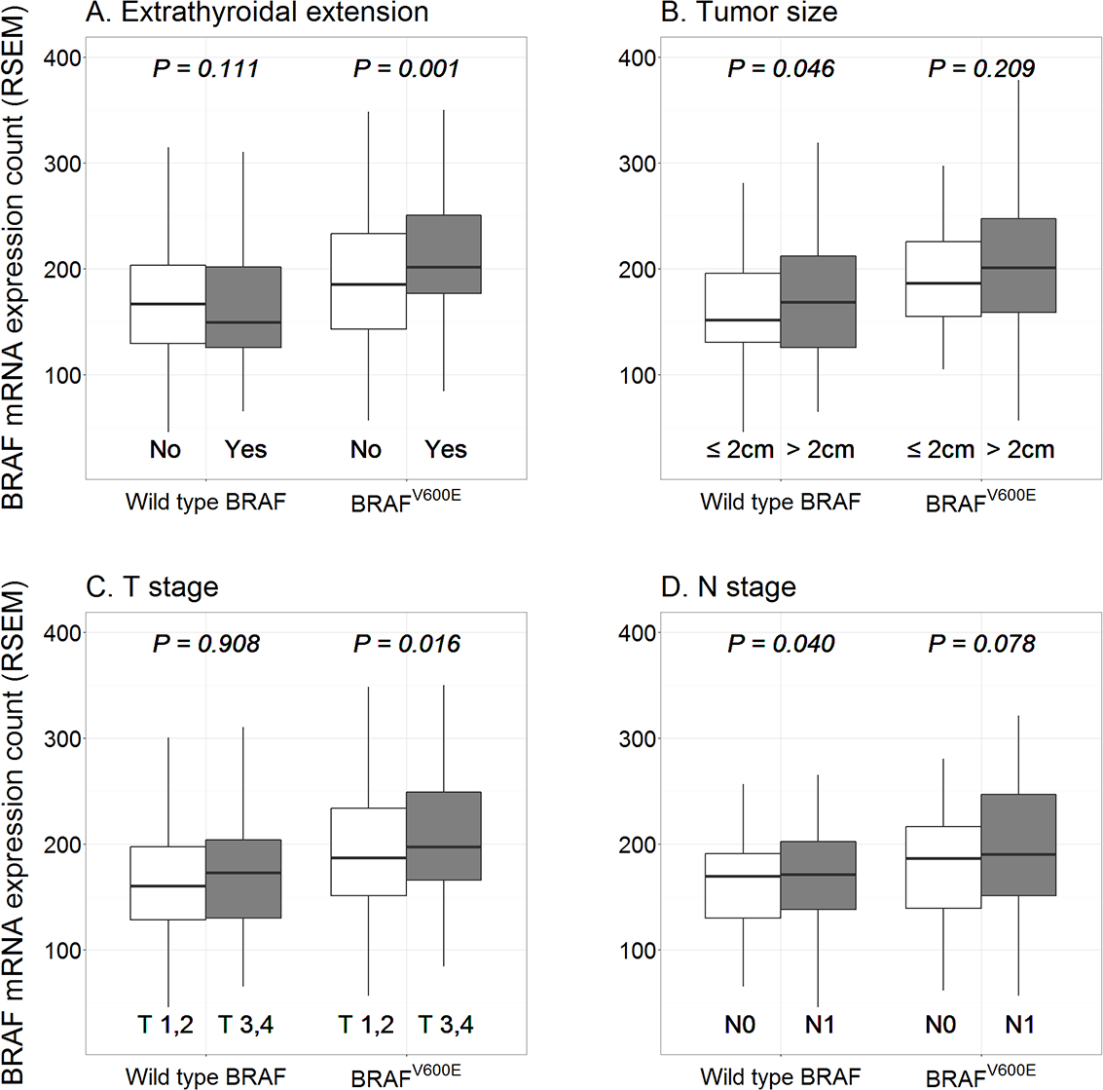
*BRAF* mRNA expression counts according to clinicopathological features. In *BRAF*^V600E^ patients, higher *BRAF* mRNA expression was associated with (A) the presence of extrathyroidal extension and (B) higher Tumor stage (T stage). In wild-type *BRAF* patients, higher *BRAF* mRNA expression was associated with (C) tumor size > 2 cm and (D) N1 stage.

Table 2 shows the results of unpaired two sample t-test. *BRAF* mRNA expression was higher in patients < 45 years and in those without thyroiditis, with a tumor size > 2.0cm, and with N1 nodal stage. In wild-type *BRAF* patients, the *BRAF* mRNA expression count was higher in cases with a tumor size > 2 cm. In addition, the count was higher in cases with N1 nodal stage. Within *BRAF*^V600E^ patients, higher *BRAF* mRNA expression was associated with the presence of ETE and higher T stage. Fig 3 summarizes the association between *BRAF* mRNA expression count and clinicopathological characteristics. Table 3 shows the results of linear regression analysis. The *BRAF* mRNA expression count was negatively correlated with age ≥ 45 years and thyroiditis, and was positively correlated with tumor size > 2 cm and N1 nodal stage. In wild-type *BRAF* patients, the *BRAF* mRNA expression count negatively correlated with age ≥ 45 years whereas it positively correlated with ETE and higher T stage in *BRAF*^V600E^ patients.

**Table 2:** Unpaired t-test results of comparisons of each clinical variable, using *BRAF* mRNA expression counts as the response variable.

	All (n = 353)		Wild-type *BRAF* (n = 160)		*BRAF*^V600E^ (n = 193)	
	Mean (RSEM)	p-value	Mean (RSEM)	p-value	Mean (RSEM)	p-value
**Age**						
< 45 years	199.046	0.023	193.7	0.051	203.5	0.207
≥ 45 years	180.224		165.5		192.1	
**Gender**						
Male	183.224	0.310	168.3	0.271	194.4	0.647
Female	191.554		183.1		198.9	
**Thyroiditis**						
No	199.195	0.032	200.8	0.104	198.0	0.162
Yes	176.810		167.0		184.0	
**Extrathyroidal extension**						
No	196.560	0.178	185.0	0.111	186.4	0.001
Yes	185.683		164.8		216.4	
**Tumor size**						
≤ 2.0 cm	179.442	0.028	163.8	0.046	191.4	0.209
> 2.0 cm	196.233		189.4		202.3	
**T stage**						
T1, T2	183.554	0.079	178.7	0.908	188.1	0.016
T3, T4	197.910		180.3		210.2	
**N stage**						
N0	175.960	0.013	157.9	0.040	188.6	0.078
N1	197.587		188.5		205.8	
**AJCC stage**						
I, II	193.133	0.157	187.3	0.073	197.9	0.934
III, IV	181.824		164.4		197.0	
**Recurrence**						
No	187.747	0.191	177.7	0.364	196.3	0.424
Yes	207.181		201.0		211.2	
**Vital Status**						
Alive	188.792	0.574	179.4	0.639	196.7	0.563
Dead	201.301		174.2		216.4	

Abbreviations: RSEM = RNA-Seq by Expectation Maximization; AJCC = American Joint Committee on Cancer

**Table 3:** Linear regression analysis of comparisons of each clinical variable, using *BRAF* mRNA expression counts as the response variable.

	All (n = 353)		Wild-type *BRAF* (n = 160)		*BRAF*^V600E^ (n = 193)	
	t-value	p-value	t-value	p-value	t-value	p-value
Age ≥ 45 years	-2.318	0.021	-2.001	0.047	-1.263	0.208
Male gender	0.908	0.365	0.909	0.365	0.448	0.655
Thyroiditis	-2.213	0.028	-1.855	0.066	-1.299	0.196
Extrathyroidal extension	1.255	0.210	-1.281	0.202	3.305	0.001
Tumor size > 2.0 cm	2.035	0.043	1.772	0.078	1.201	0.231
T stage (T3, T4)	1.727	0.085	0.108	0.914	2.462	0.015
N stage (N1)	2.314	0.021	1.839	0.068	1.685	0.094
AJCC stage (III, IV)	-1.314	0.190	-1.538	0.126	-0.086	0.932
Recurrence	1.288	0.199	0.83	0.408	0.935	0.351
Mortality	0.598	0.551	-0.129	0.897	0.921	0.358

Abbreviations: RSEM = RNA-Seq by Expectation Maximization; AJCC = American Joint Committee on Cancer

## Discussion

Since the start of the TCGA project in 2006, multiplatform genomic data from more than 20 types of carcinomas have been provided through the TCGA data portal, and multiple studies have been performed using this powerful open resource. Several studies of thyroid carcinoma using this data resource have been reported recently [14, 17, 18]. The TCGA portal provides an enormous amount of information, including data regarding microsatellite instability, DNA sequencing, miRNA sequencing, protein expression, mRNA sequencing, DNA methylation, copy number variation, clinical information, and clinical images. Although TCGA genomic data are archived under standardized and strictly controlled condition, some concerns remain regarding the correlation of genomic data with clinical information due to the potential inaccuracy of the clinical information. Such potential discrepancies exist because the surgical procedures and pathologic reporting systems differed among the institutes that collected the data. Therefore, to ensure the reliability of the TCGA data, we examined each scanned original pathologic file and revised the clinical information. Moreover, to examine the reliability of the TCGA data, we performed survival analysis; the results showed that overall survival was associated with older patient age, absence of thyroiditis, higher T stage, and higher AJCC stage, consistent with the known relationships between these parameters and overall survival in PTC.

The MAPK pathway [Ras Raf MEK MAPK/ERK] is a fundamental intracellular signaling pathway that plays a central role in cellular functions such as proliferation, differentiation, apoptosis, and survival [19]. Several mutations in the MAPK pathway are involved in the tumorigenesis of PTC. Of these, the *BRAF*^V600E^ mutation is the most potent activator of the MAPK pathway [20]. The *BRAF*^V600E^ kinase is more active than the wild-type *BRAF*, and its transformation efficacy is 138- to 500-fold higher [21, 22]. The clinical impact of the *BRAF*^V600E^ mutation is well known, but the significance of the *BRAF* mRNA expression level has not been previously studied well.

Although there are concerns that the mRNA expression level may not provide a true reflection of the protein expression level, RNA sequencing has the advantage in that the results obtained using this method can be objectively measured and there are studies reporting positive correlation between the mRNA expression level and protein expression level. One study showed that differentially expressed mRNA correlated well with their protein levels [23] and another study showed that more than 85% of the variation in steady-state protein levels could be explained by changes in mRNA levels [24]. In this regard, we used the *BRAF* mRNA expression level as a surrogate marker for Braf function in thyroid carcinoma, although a substantial proportion of variation in the protein level cannot be explained wholly by changes in the mRNA level alone [25].

The significance of *BRAF* mRNA expression in thyroid tumors has been reported previously [26], and recurrence or distant metastasis in PTC patients has been shown to be related to higher *BRAF* mRNA expression [26]. In the present study, we found an association between the *BRAF* mRNA expression level and clinicopathological characteristics in a large number of cases. We found that higher *BRAF* mRNA expression levels were associated with aggressive clinical features in both wild-type *BRAF* and *BRAF*^V600E^ patients except patient age < 45 years which is controversial as a cutoff for favorable prognosis [27]. On the other hand, we observed no differences in the clinicopathological characteristics of wild-type *BRAF* and *BRAF*^V600E^ patients, which is in contrast to the results of other studies showing that the *BRAF*^V600E^ mutation is associated with poor prognostic factors [28]. This might be because a relatively small number of patients with short term follow-up weakened the statistical power. On the other hand, it can be also postulated that the expression level of *BRAF* mRNA contributes to aggressiveness of PTC, and PTC with *BRAF*^V600E^ may not exhibit aggressive features unless mRNA expression of the *BRAF*^V600E^ reaches a certain level. Additionally, the present dataset might have included relatively low numbers of PTCs with high *BRAF*^V600E^ mRNA expression counts, which would have prevented observing a statistical difference in clinicopathological features between wild-type *BRAF* and *BRAF*^V600E^ patients. In fact, a number of studies with small sample size could not demonstrate that *BRAF* mutation is a poor prognostic factor [11, 12, 29, 30], whereas studies with larger sample sizes could [8, 31].

The significance of the Braf protein expression level has been demonstrated in previous studies using immunohistochemistry. Immunohistochemical studies suggest that higher Braf protein expression is associated with poor prognostic factors in PTC and melanoma. Zagzag et al. [13] performed an immunohistochemical study using a mutation-specific antibody (VE1; Springer-Bio, Maisons-Alfort Cedex, France) and reported that higher Braf^V600E^ mutant protein expression is associated with the presence of ETE in PTC. Although immunohistochemical studies are difficult to evaluate objectively or quantitatively, and the results of such studies are sometimes not reproducible [32], detection of Braf^V600E^ mutant protein using a mutation-specific antibody has been validated in several studies [33–35]. An association between high Braf expression and adverse prognostic outcomes was also demonstrated in melanoma [36].

The possible heterogeneous distribution of the *BRAF* mutation in PTC tumors is an important consideration in interpreting *BRAF* mRNA expression. Whether the *BRAF* mutation in PTC is clonal or subclonal has been the subject of much debate. Up until recently, the general perception was that *BRAF* mutations in PTC are strong driver mutations and PTCs with the *BRAF* mutation are clonal. This viewpoint is supported by the results of an immunohistochemical study using a Braf^V600E^ mutation-specific antibody [37] as well by a recent publication from the TCGA Research Network [17]. Conversely, a number of recent studies suggest that tumors are heterogeneous for the *BRAF* mutation and that not every cell in a *BRAF* mutation-positive tumor has a heterozygous mutation [14, 15, 38–40]. These studies also demonstrated that the percentage of *BRAF* mutant alleles in PTC with the *BRAF*^V600E^ mutation were significantly below 50%, the theoretical percentage for a clonal heterozygous mutation [14, 38–40]. Additionally, Guerra et al. subsequently demonstrated that a higher percentage of *BRAF* mutant alleles are associated with older age, larger tumor sizes, and higher recurrence rates in PTC [15]. Other studies demonstrated that a higher proportion of *BRAF* mutant cells in PTC is associated with larger tumor size [40, 41] and lymph node metastasis [40]. Likewise, the present study showed that high *BRAF* mRNA expression correlates with aggressive clinical features of PTC, probably because the tumors with high *BRAF* mRNA expression had a greater number of *BRAF*^V600E^ mutant cells. Further molecular investigations, including single cell sequencing, might help resolve the heterogeneity issue. If the *BRAF* mutation proves to be heterogeneous in PTC, it may turn out that PTCs cannot be categorized merely on whether they are *BRAF* mutated or not.

Regarding the role of *BRAF* mRNA expression in wild-type PTC, the issue of tumor heterogeneity may have little impact on the aggressive clinicopathological characteristics. There may be increased activities of the Braf kinase in association with presumptive increased Braf protein levels as a result of the overexpression of wild-type *BRAF* mRNA in aggressive PTC compared with indolent PTC. Consequently, the downstream signaling activities of the MAP kinase pathway may be more active in aggressive PTC than in indolent PTC; this would be consistent with the fact that overexpression of wild-type *BRAF* also causes increased activation of downstream signaling of the MAP kinase pathway [42–44]. This may explain the finding in the present study that *BRAF* mRNA overexpression was associated with aggressive features even in PTC with the wild-type *BRAF*. The clinical significance of this finding needs further studies to define. It is also interesting that in this study the mean *BRAF* mRNA expression level was significantly higher in PTC with *BRAF*^V600E^ than in PTC with the wild-type *BRAF* although there was no significant difference in the tumor aggressiveness between the two groups. The roles of the wild-type *BRAF* and *BRAF*^V600E^ in tumor progression are apparently complex. Further mechanistic studies are needed to fully understand these findings.

Meanwhile, various *BRAF* inhibitors are currently being tested in clinical trials on patients with refractory thyroid carcinoma, with varying results. Subclonality, splice variants, or copy number gains of *BRAF* may be responsible for the lack of responses to *BRAF* inhibitors in many cases [45–47]. In selecting patients who may benefit from *BRAF* inhibitors, the *BRAF* mRNA expression level might serve as a potentially useful selection indicator if, in the future, it is proven to be specifically correlated with the response rate to *BRAF* inhibitors. As such, *BRAF* mRNA expression level might be helpful in predicting therapeutic effects. Similarly, it could also help exclude patients who would likely not respond to unnecessary treatments with *BRAF* inhibitors, and thereby avoid unnecessary exposure of such patients to the undesirable side effects of these drugs.

In conclusion, high *BRAF* mRNA expression levels were associated with aggressive clinical features in both wild-type *BRAF* and *BRAF* ^V600E^ patients with classic PTC, although the association with recurrence or survival could not be shown due to limited follow-up. Although heterogeneity of the *BRAF* mutation could weaken the correlation between the *BRAF* mRNA expression level and aggressive clinical features, the correlation was probably more robust in the PTC samples with higher percentages of *BRAF* mutant cells. Evaluation of the *BRAF* mRNA expression level in patients could provide a way to establish more accurate prognoses and risk stratification.

## Supporting Information

S1 Table Clinicopathological features and BRAF mRNA expression counts of the study patients. (XLSX)
